# *Cassia spectabilis* (DC) Irwin et Barn: A Promising Traditional Herb in Health Improvement

**DOI:** 10.3390/molecules170910292

**Published:** 2012-08-29

**Authors:** Subramanion L. Jothy, Angeline Torey, Ibrahim Darah, Yee Siew Choong, Dharmaraj Saravanan, Yeng Chen, Lachimanan Yoga Latha, Subramanian Deivanai, Sreenivasan Sasidharan

**Affiliations:** 1 Institute for Research in Molecular Medicine (INFORMM), Universiti Sains Malaysia, 11800 USM, Penang, Malaysia; Email: psiqueangelus@yahoo.com (A.T.); yeesiew@usm.my (Y.S.C.); latha_usm@yahoo.com (L.Y.L.); 2 School of Biological Sciences, Universiti Sains Malaysia, 11800 USM, Penang, Malaysia; Email: darah@usm.my; 3 Faculty of Medicine and Health Sciences, Universiti Sultan Zainal Abidin, Kota Kampus, 20400 Kuala Terengganu, Terengganu, Malaysia; Email: saravanandharmaraj@yahoo.com; 4 Dental Research and Training Unit, and Oral Cancer Research and Coordinating Centre (OCRCC), Faculty of Dentistry, University of Malaya, Kuala Lumpur 50603, Malaysia; Email: chenyeng@um.edu.my; 5 Department of Biotechnology, Faculty of Applied Sciences, AIMST University, Jalan Bedong Semeling, Batu 3½, Bukit Air Nasi, Bedong 08100, Kedah, Malaysia; Email: deivanai_subramanian@yahoo.com.sg

**Keywords:** *Cassia spectabilis*, pharmacological properties, ethnomedicinal uses, phytochemistry

## Abstract

The genus *Cassia*, comprising about 600 species widely distributed worldwide is well known for its diverse biological and pharmacological properties. *Cassia spectabilis* (sin *Senna spectabilis*) (DC) Irwin et Barn (Fabaceae) is widely grown as an ornamental plant in tropical and subtropical areas. *C. spectabilis* has been commonly used in traditional medicine for many years. Information in the biomedical literature has indicated the presence of a variety of medicinally-important chemical constituents in *C. spectabilis*. Pharmacological studies by various groups of investigators have shown that *C. spectabilis* possesses significant biological activity, such as antibacterial, antibiofilm, antifungal and antioxidant properties. Beside this, toxicity studies of this plant have revealed no toxic effect on mice. In view of the immense medicinal importance of *C. spectabilis*, this review aimed at compiling all currently available information on *C. spectabilis*’s botany, phytochemistry, pharmacology, and mechanism of actions, toxicology and its ethnomedicinal uses.

## 1. Introduction

**Figure 1:**
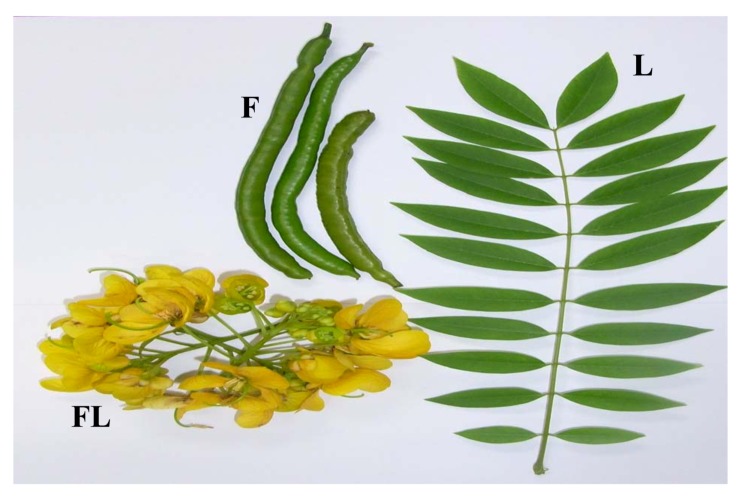
*Cassia spectabilis.* F, fruit; FL, flower and L, leaf.

Plants are the “ancient friend” of mankind. Without plants there is would be no humans on Earth because we depend entirely on the oxygen produced by plants during photosynthesis. Moreover plants also help mankind to sustain its health by supplying phythochemicals with curative value through various foodstuffs and herbal remedies. Hence it is important to investigate medicinal plants comprehensively in order to enhance our knowledge of these plants to assist in the development of pharmaceutical products. Moreover only a limited number of medicinal plants have received detailed scientific scrutiny, thereby prompting the World Health Organization to recommend that this area could be comprehensively investigated [1]. Plants used in traditional medicine contain a wide range of substances that can be used to treat chronic as well as infectious diseases [2]. One such plant belonging to the genus *Cassia* and known to have curative value is *Cassia spectabilis* (sin *Senna spectabilis*) (DC) Irwin et Barn (Fabaceae) (Figure 1). The genus *Cassia*, comprising about 600 species widely distributed worldwide is well known for its diverse biological and pharmacological properties [3]. In addition, *C. spectabilis* has been of medical interest due to its good therapeutic value in folk medicine. *C. spectabilis* is widely grown as an ornamental plant in tropical and subtropical areas. In light of this importance, this review focuses on the botany, phytochemistry, pharmacology, mechanism(s) of action, toxicology and ethnomedicinal uses of *C. spectabilis*.

## 2. Botany

### 2.1. Scientific Name

*Cassia spectabilis (DC.) Irwin & Barneby* (Figure 1).

### 2.2. Common Names

Cassia, mwenu, mhomba, antsoan dilaw, scented shower and Panama-ngu.

### 2.3. Synonyms

*Cassia trinitatis, Cathartocarpus trinitatis, Cassia humboldtiana, Cassia totonaca, Cathartocarpus speciosus, Cassia speciosa, Cathartocarpus humboldtianus, Cassia edulis, Cassia carnaval, Cassia amazonica, Pseudocassia spectabilis, Cassia excelsa var. Acutifolia.*

### 2.4. Classification



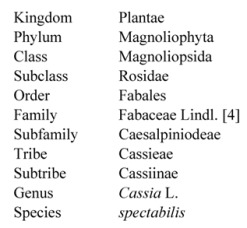



### 2.5. Distribution

*Cassia* is native to Central America and the Northern regions of South America. It has also been introduced to Africa, including Angola, Burundi, South Africa and to East Africa, as an ornamental.

### 2.6. Botanical Description

A medium to large tree, it reaches a height of 4.5–8 m with a spreading canopy of 15 to 20 feet (4.5–6 m). Leaves are alternate pinnately compound with 7–15 pairs of leaflets, which are up to 3 inches long with fuzzy undersides. Leaflets usually have pointed tips. The flowers are bright yellow in color, about 1.5 inches wide, appearing in dense racemes up to two feet long above the foliage. Inflorescences are large, terminal, lateral, leafy panicles, 15–30 cm long, which are branched and very large. Flowers are many, fragrant, composed of five rounded hairy bracts, which are ovate, 4–5 mm long, caducous; pedicles 2–3 mm, velutinous. Sepals are orange-yellow, unequal, ovate to circular, 5–7 mm long. Petals are yellow, spoon shaped, unequal, broadly to narrowly obovate, 2–3.5 cm long, anthers opening by apical pores and a slit. Stamens are seven large and three small, sterile. Pistil slender, curved, hairless. Ovary is glabrous, recurved; style and stigma inconspicuous. Fruits are long cylindrical or flattened pods, initially green in color later becomes dark or black on ripening. Pods ends in a short, narrow point, hard, none splitting. Seeds are flattened, brown and about 5 mm in diameter [5].

### 2.7. Propagation

*Cassia* can be grown by seeds but must be scarified. The seeds can remain viable for several years in storage.

### 2.8. Ethnomedicinal Uses

This *Cassia* species has been used in Asia and many other countries to treat diverse ailments. based on its antimicrobial, laxative, antiulcerogenic, analgesic, and anti-inflammatory properties [3]. In northeast Brazilian folk medicine, it has been used as an anti-inflammatory, analgesic, laxative, purgative, antimicrobial and antiulcerogenic [3,6,7]. It has also been used in traditional Brazilian medicine for the treatment of flu and colds, as a laxative and purgative [8,9]. In the Ayurvedic system of medicine these plants were also used for the treatment of fevers and headaches. Moreover, in Thai traditional medicine the plant was used for ringworm and skin diseases. *C. spectabilis* leaves are also reported as a traditional medicine to inhibit oedema, and for the treatment of constipation, poisoning and protozoic infections of the gut [1].

### 2.9. Phytochemistry

Phytochemical studies of *Cassia spectabilis* have been conducted since the mid 1970s and one of the early studies [10] reported the isolation of two alkaloids—(+)-spectaline and (−)-iso-6-cassine—from the leaves of this plant. These piperidin 3-ol alkaloids were reported for the first time. These compounds were also obtained later from the seeds, together with another two alkaloids, which were characterized as (−)-spectalinine and (−) iso-6-carnavaline [11]. Two other researchers working on aerial parts of this plant isolated another two alkaloids, identified as 2-decacylacetyl-5-hydroxy-6-methylpiperidine and 2-dodecylacetyl-5-hydroxy-6-methylpiperidine. The latter alkaloid was isolated for the first time and also named cassinicine. Four other compounds obtained from this work were β-sitosterol, stigmasterol, 1,3,8-trihydroxy-2-methylanthraquinone and 1,8-dihydroxy-3-methyl-6-methoxyanthraquinone, also called physcion [12].

**Figure 2:**
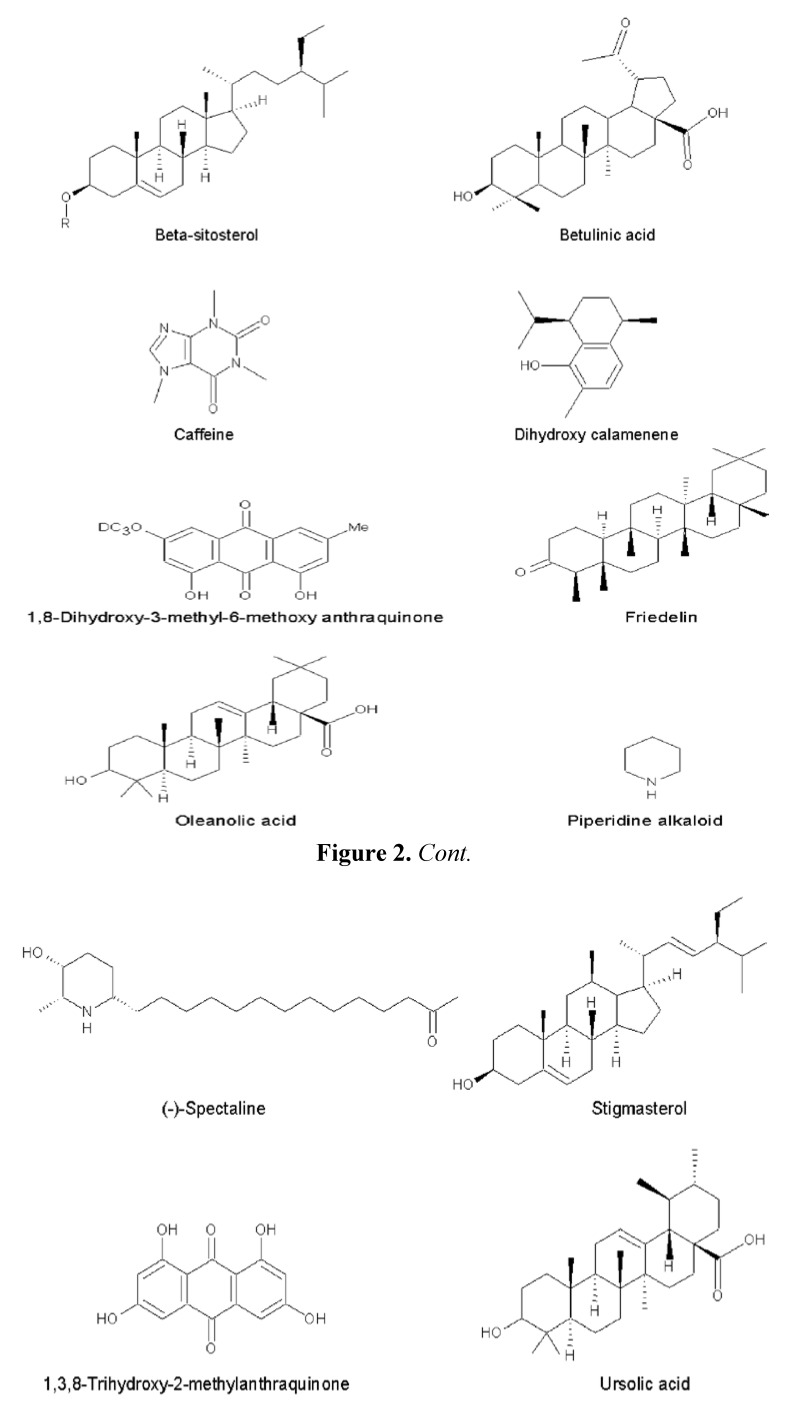
Various phytochemicals isolated from *Cassia spectabilis*.

Another group of researchers [3] used the flowers and green fruits of this plant for isolation of bioactive compounds. They obtained from the flowers three new piperidine alkaloids which were (−)-3-*O*-acetylspectaline, (−)-7-hydroxyspectaline and iso-6-spectaline, as well as the then known compound (−)-spectaline (Figure 2). The compounds (−)-spectaline and (−)-3-*O*-acetylspectaline were also found in green fruits. The DNA-damaging activity of these compounds were evaluated using a mutant yeast (*Saccharomyces cerevisiae*) assay and among the four compounds studied the compounds (−)-3-*O*-acetylspectaline and (−)-spectaline showed positive activity. This activity for (−)-spectaline was also reported by earlier researchers who isolated the compound initially from *C. leptophylla* [13]. Another study reported that (−)-spectaline possessed antinociceptive effects in the acetic acid-induced writhing test with mice. However, it did not show this effect with the tail flick or hot-plate method [14]. Further work was done to assess the antinociceptive effects of natural and semi-synthetic spectalines using the acetic acid-induced writhing test. The compound (−)-spectaline isolated from *C. spectabilis* showed the best antinociceptive effect, with (−)-3-*O*-acetylspectaline having slightly less activity. Moreover, both these natural compounds were better than synthetic derivatives [15].

Earlier, the above group of researchers isolated a new piperidine compound, (+)-3-*O*-feruloylcassine which together with (−)-spectaline and (−)-3-*O*-acetylspectaline were tested for bioactivity. All three compounds showed inhibition of lipid peroxidation with the new compound displaying less inhibition than the other two, but all three compounds were less active than butylated hydroxytoluene (BHT). The effect of these compounds on inhibition of cyclooxygenase-1 was moderate, but only marginal effects were seen on cyclooxygenase-2 [16]. 

Many piperidine alkaloids were mentioned in the above studies but the presence of only two anthraquinones were reported only in the study by Mulchaldani and Hassarajani [12]. This is surprising as the genera are reported to be rich in anthraquinones [17,18]. In fact, various studies have isolated these compounds from other species in the genus *Cassia*. For example, the study by Kim *et al.* [19] using seeds of *C. tora* reported the presence of the following anthraquinones: emodin, aloe-emodin, rhein and physcion. Another study on seeds of *C. obtusifolia* reported the presence of emodin [20]. The isolation of emodin alone [21] or emodin and alaternin or 2-hydroxyemodin [22] from *C. tora* has been reported.

The above trend with certain researchers reporting certain families of compounds in phytochemical studies is quite common. It has been mentioned that the isolation and identification of natural products is a very laborious procedure, especially considering the complexity of mixture and presence of the compounds in minute concentrations [23]. Moreover, certain groups might have been working on isolation of certain class of compounds based on activity instead of presence of all compounds in the plants. One approach to overcome this problem would be the use of electrospray ionization mass spectrometry as used by Pivatto *et al.* [24] to obtain the chemical profile or fingerprint of *C. spectabilis* extracts. Although, this group worked on alkaloid profile, the method could be used for different classes of compounds. De Oliveira Silva *et al.* [25] studied the leaves of *Senna spectabilis*, reporting the presence of the following compounds: caffeine, the triterpenes lupeol, α-amyrin, β-amyrin, cycloeucalenol, friedelin and ursolic, oleanolic and betulinic acids, besides the steroids sitosterol and stigmasterol and their respective glucosides. Overall there is still scope for further work concerning phytochemical content of *C. spectabilis*. Figure 2 shows the major compounds isolated from *C. spectabilis*.

## 3. Pharmacological Activities of *C. spectabilis*

### 3.1. Standardizations of *C. spectabilis* Leaf

Standardizations of *C. spectabilis* leaf was reported by Torey *et al.* [26] with respect to authenticity, assay and chemical constituent analysis. The authentication was done by them using many parameters, including gross morphology, microscopy of the leaves and functional group analysis by Fourier Transform Infrared (FTIR) spectroscopy. The assay part of the standardization involved determination of the minimum inhibitory concentration (MIC) of the extract against *Candida albicans.* which could help assess the chemical effects and establish curative values. The MIC of the *C. spectabilis* leaf extracts was investigated using the Broth Dilution Method. The extracts showed a MIC value of 6.25 mg/mL, independent of the extraction time. The chemical constituent aspect of the standardization involved quantification of the main chemical components in *C. spectabilis*. The GC-MS method used for quantification of 2,4-(1*H*,3*H*)-pyrimidinedione in the extract was rapid, accurate, precise, linear (R^2^ = 0.8685), rugged and robust. Hence they reported that this method was suitable for quantification of this component in *C. spectabilis*. The also reported that the standardization of *C. spectabilis* was important to facilitate marketing of this plants, with a view to promoting the export of valuable Malaysian traditional medicinal plants.

### 3.2. Antibiofilm Activity

Preliminary studies on the effects of *Cassia spectabilis* leaf extract on *C. albicans* biofilm were reported by Sangetha *et al.* [27] and they were evaluated using scanning electron microscopy (SEM). Visualization of the ultrastructure in general revealed a marked reduction in biofilm and also a reduction in adhering cells for cells that were treated with *C. spectabilis* leaf extract for 36 h compared to the untreated ones. This suggests that the leaf extract of *C. spectabilis* may exert a metabolic interference in the biofilm. As biofilm formation and development involve a series of steps, cell responses and interactions, any intervention of these steps may possibly inhibit its formation.

Apart from that, Torey and Sasidharan [28] recently reported the detailed antibiofilm activity of *C. spectabilis* leaf extract. In this study *C. spectabilis* leaves extract was assessed using the 2,3-bis [2-methoxy-4-nitro-5-sulphophenyl]-2*H*-tetrazolium-5-carboxanilide (XTT) reduction assay for biofilm quantification with positive control fluconazole. Scanning electron microscopic (SEM) and confocal scanning laser microscopy (CLSM) analysis revealed reduction in *C. albicans* biofilm by *C. spectabilis* leaves extract. They also reported that the methanol extract of *C. spectabilis* showed a favorable anti-yeast activity against *C. albicans* with a MIC value of 6.25 mg/mL. Fluconazole and leaves extract showed 95.4% and 96.9% biofilm reduction, respectively. The main changes observed under scanning electron microscopy after *C. spectabilis* leaves extract treatment were cellular damage and disruption in biofilms of *C. albicans*, which was further confirmed using confocal scanning laser microscopy (CLSM).

### 3.3. Antifungal Activity

The methanolic extracts of the *C. spectabilis* leaves, flowers, stem and pods were evaluated for their antifungal activity against *Saccharomyces cerevisiae* and *Aspergillus niger* [29] using the disk diffusion assay. *Aspergillus niger* was susceptible to all extracts of *Cassia spectabilis*. The leaf, flower, stem and pod extracts showed inhibition zones of 12 mm, 12 mm, 7 mm and 11 mm, respectively, but they were lower than the positive control miconazole nitrate (30 µg/mL) which had an inhibition zone of 25 mm. However, *Saccharomyces cerevisiae* was resistant to all the extracts tested. In another study by Sangetha *et al.* [30], the antifungal activity of the *C. spectabilis* leaf extract on *Candida albicans* was studied and the zone of inhibition obtained was 16 mm, compared to miconazole nitrate (30 µg/mL) which had a zone of 21 mm.

In addition to the disk diffusion method, the *C. spectabilis* leaf extract was also subjected to the broth dilution method to determine the minimum inhibitory concentration (MIC) on *Candida albicans* [30]. The extract showed a favourable antifungal activity, with a MIC of 6.25 mg/mL. Besides that, *in vivo* studies were carried out as well by treating mice with 2.5 g/kg body weight dose of the leaf extract and subsequently enumerating the colony forming units (CFU). There was a 5-fold reduction in the CFU/g of organ and CFU/mL of blood when compared to the untreated controls. Further studies on the *Candida albicans* morphology were also carried out using scanning electron microscopy (SEM) and transmission electron microscopy (TEM). After 24 h of treatment, the SEM observation showed that the cells had a rough appearance, with the formation of invaginations compared to the untreated control cells. After treatment for 36 h, the cells were completely collapsed and appeared cavitated and it was believed that at this stage, the cells had completely lost their metabolic functions. As for the TEM observation, after 24 h of exposure to the extract, the cells exhibited notable alterations in the cell membrane and the cell wall. After treatment for 36 h, the yeast cells were found to be collapsed and lysed, followed by an outflow of the cytoplasmic components. These results suggested that it is possible that the *C. spectabilis* leaf extract acts on the cell wall or cell membrane of the *C. albicans* cells, thus arresting their growth.

Silva *et al.* [31] isolated and identified five cadinane sesquiterpenes derivatives by bioassay-guided fractionation from *Phomopis cassiae*, an endophytic fungus isolated from *Cassia spectabilis*. The structures of the two diastereoisomeric 3,9,12-trihydroxycalamenenes; 3,12-dihydroxycalamenene; 3,12-dihydroxycadalene and 3,11,12-trihydroxycadalene were established on the basis of analyses of 1D and 2D NMR and HRTOFMS experiments. They evaluated antifungal activity against *Cladosporium sphaerospermum* and *Cladosporium cladosporioides* and the results revealed 3,11,12-trihydroxycadalene as the most active compound.

### 3.4. Antibacterial Activity

The disk diffusion assay and the minimum inhibitory concentration (MIC) assay using serial tube dilution technique were employed in the study by Sangetha *et al.* [29] to investigate the antibacterial potency of *C. spectabilis*. The bacteria studied included *Proteus mirabilis*, *Staphylococcus aureus*, *Bacillus thuringiensis*, *Escherichia coli*, *Salmonella typhi*, *Micrococcus sp.*, *Enterobacter aerogenes*, *Bacillus subtilis*, *Azospirilium lipoferum*, *Klebsiella pneumoniae* and *Pseudomonas aeruginosa.* Overall, the leaf, flower, stem and pod extracts showed significant antibacterial activity against both Gram-positive and Gram-negative bacteria when compared to chloramphenicol which was used as a positive control. The *C. spectabilis* leaf extract was the most active one and it inhibited the growth of all the bacterial strains tested, specifically *Micrococcus sp.* (35 mm), *Staphylococcus aureus* (30 mm) and *Bacillus subtilis* (30 mm). As for the MIC assay, the MIC values against these Gram-positive and Gram-negative bacteria ranged from 0.195 to 50.000 mg/mL. The MIC results also indicated that the leaf extract is effective against Gram-positive bacteria at a lower concentration (0.195 mg/mL for *Bacillus subtilis*) compared to Gram-negative bacteria (50.000 mg/mL for *Pseudomonas aeruginosa*).

Furthermore, Krishnan *et al.* [32] also reported the antimicrobial properties of various extracts namely acetone, *n*-hexane, dichloromethane, ethyl acetate and methanol leaf extract against Gram positive bacteria (*Bacillus subtilis* and *Staphylococcus aureus*) and Gram negative bacteria (*Escherichia coli*, *Salmonella typhi* and *Pseudomonas aeroginosa*). They determined the MIC, and minimum bactericidal concentration (MBC) by using a microdilution assay. In their study the methanol extract showed the highest yield (14.12%) followed by dichloromethane (8.37%), acetone (6.66%), ethyl acetate (4.76%) and *n*-hexane (1.80%). They also reported that the acetone and methanol extracts showed good antimicrobial activit, with MIC values ranging from 0.625 to 2.5 mg/mL and MBC values ranging from 1.25 to 5 mg/mL. The MIC and MBC values of these extracts were 10 to 80 times less potent than standard antimicrobial drugs, amoxicillin and miconazole nitrate they used in their study.

### 3.5. Antioxidant Activity

The antioxidant activity of *Cassia spectabilis* was evaluated by Sangetha *et al.* [33] using the 2,2-diphenyl-1-picrylhydrazyl (DPPH) radical-scavenging assay. It was reported that the flower, stem, leaf and pod extracts exhibited 54.29%, 53.28%, 45.17% and 6.18% of radical-scavenging activities, respectively, at 1.0 mg/mL of extract tested.

### 3.6. Anti-inflammatory and Anti-hyperalgesics Activity

The anti-nociceptive and anti-inflammatory effects of (−)-cassine, isolated from *Senna spectabilis* were evaluated using pharmacological, behavioural and biochemical approaches by da Silva *et al.* [33]. The results of their work demonstrated a pronounced anti-inflammatory and anti-nociceptive properties of this alkaloid. Its anti-inflammatory and anti-nociceptive properties are likely to be associated with its ability to inhibit the activation and/or release of various inflammatory mediators such as KC, IL-1β and IL-6. Also, the anti-nociceptive effects of this compound seem to be closely associated with its marked inhibition of PGE_2_ activity [34].

### 3.7. Anticonvulsant Activities

Bum *et al.* [35] reported the anticonvulsant and sedative properties of *S. spectabilis* in mice, which could explain the use of this plant in traditional medicine in Africa, particularly in Cameroon, in the treatment of insomnia and epilepsy. Meanwhile, Silva *et al.* [36] also reported the depressant and anticonvulsant activities of iso-6-cassine (ISO), another compound isolated from *S. spectabilis*. They reported that by administered the isolated compound at 0.5, 1.0 and 1.5 mg/kg concentration by oral route in mice caused a significant decrease in the motor activity of animals when compared with the control group, up to 30 days after the administration and at dose of 1.5 mg/kg, it reduced the permanence time of animals on a Rota-rod apparatus.

## 4. Dosage/Mode of Usage

Flower, leaf, root and pod can be used as potential herbal samples in pharmacy as decoction. 

## 5. Toxicological Assessment

No adverse effects have been reported for the usage of *C. spectabilis* as a drug. Sangetha *et al.* [29] conducted a cytotoxicity assay on brine shrimp nauplii using a method described by Meyer [37]. The LC_50_ value obtained for the *C. spectabilis* methanol leaf extract was 2.20 mg/mL. The cytotoxicity of the plant extract was compared to the cytotoxicity of potassium dichromate which was used as a standard, whose LC_50_ value was 0.41 mg/mL. Besides that, a linear correlation was observed when logarithm of concentration *versus* percentage of mortality was plotted on a graph. The results for the brine shrimp assay indicated that the extract has an LC_50_ value greater than 1.0 mg/mL which is the recommended cutoff point for detecting cytotoxic activity. This suggested that this plant may not be toxic.

Furthermore, an oral acute toxicity study was also carried out on mice by Sangetha *et al.* [30] for further toxicological assessment and no toxic symptoms or death was observed in the animals and all of them lived up to 14 days. An autopsy at the end of the experimental period revealed no apparent changes in any organs; there were also no changes in either the body weight or the weight of the principal organs and all animals exhibited a gain in body weight. Therefore, the acute minimum fatal dose of *C. spectabilis* leaf extract for mice was over 2,000 mg/kg body weight which is not toxic according to OECD guidelines [38].

## 6. Precautions/Safety for Usage

The safety of medicinal plants is of major importance since many herbal plants are self prescribed and patients usually do not inform their doctors that herbal medicines are being consumed. Scientific information on the safety and effective usage *C. spectabilis* is hard to find. *C. spectabilis* is normally found on road sides. Hence, precautions should be taken to ensure collection of herb that has not been sprayed with weed killer. The samples are to be washed thoroughly or soaked with water to remove unwanted pollution. In addition, patients who are taking herbs concurrently with commercially available medication have to be very careful because it interacts with commercial medicine and should inform their doctors that herbal medicines are being taken.

## 7. Conclusions

In this review, we attempted to collect the botanical, phytochemistry, pharmacological, mechanism of actions, toxicological and ethnomedicinal information on *C. spectabilis*, a medicinal herb species used in the traditional medicine and a potential medicinal plant for the modern era. The survey of the literature revealed the presence of alkaloids in *C. spectabilis*. It also revealed a broad spectrum of pharmacological activity of the species.

## References

[1] Mazumder P.M., Percha V., Farswan M., Upaganlawar A. (2008). *Cassia*: A Wonder Gift to Medical Sciences. Int. J. Clin. Pharm..

[2] Duraipandiyan V., Ayyanar M., Ignacimuthu S. (2006). Antimicrobial activity of some ethnomedicinal plants used by Paliyar tribe from Tamil Nadu, India. BMC Complement. Altern. Med..

[3] Viegas C., Bolzani V.S., Furlan M., Furlan M.S., Barreiro E.J., Young M.C.M., Tomazela D., Eberlin M.N. (2004). Further bioactive piperidine alkaloids from the flowers and green fruits of *Cassia spectabilis*. J. Nat. Prod..

[4] Lindley J. (1830). Report upon the New or Rare Plants which Flowered in the Garden of the Horticultural Society at Chiswick, between March, 1825, and March, 1826, Part 1.

[5] AgroForestry Tree Datadase A Tree Species Reference and Selection Guide.

[6] Sansores-Peraza P., Rosado-Valladi M., Brito-Loeza W., Mena-Rejon G.J., Quijano L. (2000). Cassine, an antimicrobial alkaloid from *Senna racemosa*. Fitoterapia.

[7] Freitas R.M., Souza F.C., Viana G.S., Fonteles M.M. (2006). Acetylcholinesterase activities in hippocampus, frontal cortex and striatum of Wistar rats after pilocarpine-induced status epilepticus. Neurosci. Lett..

[8] Lorenzi H., de Abreu Matos F.J. (2002). Plantas Medicinais no Brasil: Nativas e Exóticas.

[9] Carlini E.A. (2003). Plants and the central nervous system. Pharmacol. Biochem. Behav..

[10] Christofidis I., Welter A., Jadot J. (1977). Spectaline and iso-6-cassine, two new piperidin 3-ol alkaloids from the leaves of *Cassia spectabilis*. Tetrahedron.

[11] Christofidis I., Welter A., Jadot J. (1977). Spectalinine and iso-6-carnavaline, two unprecedented piperidine alkaloids from the seeds of *Cassia spectabilis*. Tetrahedron.

[12] Mulchaldani N.B., Hassarajani S.A. (1977). Cassinicine, a new alkaloid and anthraquinones from *Cassia spectabilis* and their biogenetic relationship. Planta Med..

[13] Bolzani V.S., Gunatilaka A.A.L., Kingston D.G.I. (1995). Bioactive and other piperidine alkaloids from *Cassia leptophylla*. Tetrahedron.

[14] Alexandre-Moreira M.S., Viegas C.Jr., de Miranda A.L.P., Bolzani V.S., Barreiro E.J. (2007). Antinociceptive profile of (−)-spectaline: A piperidine alkaloid from *Cassia leptophylla*. Planta Med..

[15] Viegas C.Jr., Alexandre-Moreira M.S., Fraga C.A.M., Barreiro E.J., Bolzani V.S,  de Miranda A.L.P. (2008). Antinociceptive profile of 2,3,6-trisubstituted piperidine alkaloids: 3-*O*-acetylspectaline and semisynthetic derivatives of (−)-spectaline. Chem. Pharm. Bull..

[16] Viegas C.Jr., Silva D.H.S., Rivatto M., de Rezende A., Castro-Gamboa I., Bolzani V.S., Nair M.G. (2007). Lipoperoxidation and cyclooxygenase enzyme inhibitory piperidine alkaloids from *Cassia spectabilis* green fruits. J. Nat. Prod..

[17] Pineyro-Lopez A., Waksman N., Atta-ur-Rahman (2000). Chemistry, Structure and Biological Activity of Anthracenones of the Karwinskia Genus. Studies in Natural Product Chemistry.

[18] Springob K., Kutchan T.M., Osbourn A.E., Lanzotti V. (2009). Introduction to different classes of natural products. Plant-Derived Natural Products.

[19] Kim Y.M., Lee C.H., Kim H.G., Lee H.S. (2004). Anthraquinones isolated from *Cassia tora*(Leguminosae) seed show an antifungal property against phytopathogenic fungi. J. Agric. Food Chem..

[20] Yang Y.C., Lim M.Y., Lee H.S. (2003). Emodin isolated from *Cassia obtusifolia* (Leguminosae) seed shows larvicidal against three mosquito species. J. Agric. Food Chem..

[21] Yen G.C., Chen H.W., Duh P.D. (1998). Extraction and identification of an antioxidative component from jue ming zi (*Cassia tora* L.). J. Agric. Food Chem..

[22] Choi J.S., Chung H.Y., Jung H.A., Park H.J., Yokozawa T. (2000). Comparative evaluation of antioxidant potential of alaternin (2-hydroxyemodin) and emodin. J. Agric. Food Chem..

[23] Calixto J.B., Santos A.R.S., Filho V.C., Yunes A.R. (1998). A review of the plants of the genus *Phyllanthus*: Their chemistry, pharmacology and therapeutic potential. Medical Res. Rev..

[24] Pivatto M., Croth A.E.M., Lopes N.P., Castro-Gamboa I., de Rezende A., Viegas C.Jr., Young M.C.M., Furlan M., Bolzani V.S. (2005). Electrospray ionization mass spectrometry screening of piperidine alkaloids from *Senna spectabilis* (Fabaceae) extracts: Fast identification of new constituents and co-metabolites. J. Braz. Chem. Soc..

[25] de Oliveira Silva F., de Oliveira I.R., de Vasconcelos Silva M.G., Braz-Filho R. (2010). Chemical compounds of leaves from *Senna spectabilis* (DC) Irwin & Barneby var. excelsa (Schrad.) Irwin & Barneby. Quimica Nova.

[26] Torey A., Sasidharan S., Yeng C., Latha L.Y. (2010). Standardization of *Cassia Spectabilis* with respect to authenticity, assay and chemical constituent analysis. Molecules.

[27] Sangetha S., Zuraini Z., Suryani S., Sasidharan S. (2009). *In situ* TEM and SEM studies on the antimicrobial activity and prevention of *Candida albicans* biofilm by *Cassia spectabilis* extract. Micron.

[28] Torey A., Sasidharan S. (2011). Anti-candida albicans biofilm activity by *Cassia Spectabilis* standardized methanol extract: An ultrastructural study. Eur. Rev. Med. Pharmacol. Sci..

[29] Sangetha S., Zuraini Z., Sasidharan S., Suryani S. (2008). Antibacterial, Antifungal and Cytotoxic Activities of *Cassia spectabilis*. Asian J. Pharm. Clin. Res..

[30] Sangetha S., Zuraini Z., Sasidharan S., Suryani S. (2008). Fungicidal Effect and Oral Acute Toxicity of *Cassia spectabilis* Leaf Extract. Nippon Ishinkin Gakkai Zasshi.

[31] Silva G.H., Teles H.L., Zanardi L.M., Young M.C.M., Eberlin M.N., Hadad R., Pfenning L.H., Costa-Neto C.M., Castro-Gamboa I., da Silva Bolzani V. (2006). Cadinane sesquiterpenoids of *Phomopsis cassiae*, an endophytic fungus associated with *Cassia spectabilis* (Leguminosae). Phytochemistry.

[32] Krishnan N., Ramanathan S., Sasidharan S., Murugaiyah V., Mansor S.M. (2010). Antimicrobial activity evaluation of *Cassia Spectabilis* leaf extracts. Int. J. Pharmacol..

[33] Sangetha S., Zuraini Z., Sasidharan S., Suryani S. (2008). Free Radical Scavenging Activity of *Cassia spectabilis* and *Cassia fistula*. Int. J. Nat. Eng. Sci..

[34] da Silva K.A., Manjavachi M.N., Paszcuk A.F., Pivatto M., Viegas C.Jr., Bolzani V.S., Calixto J.B. (2012). Plant derived alkaloid (−)-cassine induces anti-inflammatory and anti-hyperalgesics effects in both acute and chronic inflammatory and neuropathic pain models. Neuropharmacology.

[35] Bum E.N., Nkantchoua G.N., Njikam N., Taiwe G.S., Ngoupaye G.T., Pelanken M.M., Nanga, Maidawa F., Rakotonirina A., Rakotonirina S.V.  (2010). Anticonvulsant and sedative activity of leaves of *Senna spectabilis* in mice. Int. J. Pharmacol..

[36] de Oliveira Silva F., de Vasconcelos Silva M.G., Feng D., de Freitas R.M.  (2011). Evaluation of central nervous system effects of iso-6-cassine isolated from *Senna spectabilis* var. *excelsa* (Schrad) in mice. Fitoterapia.

[37] Meyer B.N., Ferrigini R.N., Jacobsen L.B., Nicholas D.E., McLaughlin J.L. (1982). Brine shrimp: A convenient general bioassay for active plant constituent. Planta Med..

[38] Organization for Economic Cooperation and Development (OECD) (2000). Guidelines for the Testing of Chemicals, Revised draft guideline: Acute oral toxicity.

